# Supplementary material for: Left Atrial Diameter in the Prediction of Thromboembolic Event and Death in Atrial Fibrillation
Source: J Clin Med. 2022 Mar 26;11(7):1838. doi: 10.3390/jcm11071838 (PMC8999165; doi:10.3390/jcm11071838)

Supplementary Data

Supplementary Figure S1. Cubic spline graph for the assessment of predictive value of LADi on thromboembolic event (A. unadjusted and B. adjusted), all-cause death (C. unadjusted and D. adjusted) treating LADi as a continuous variable.

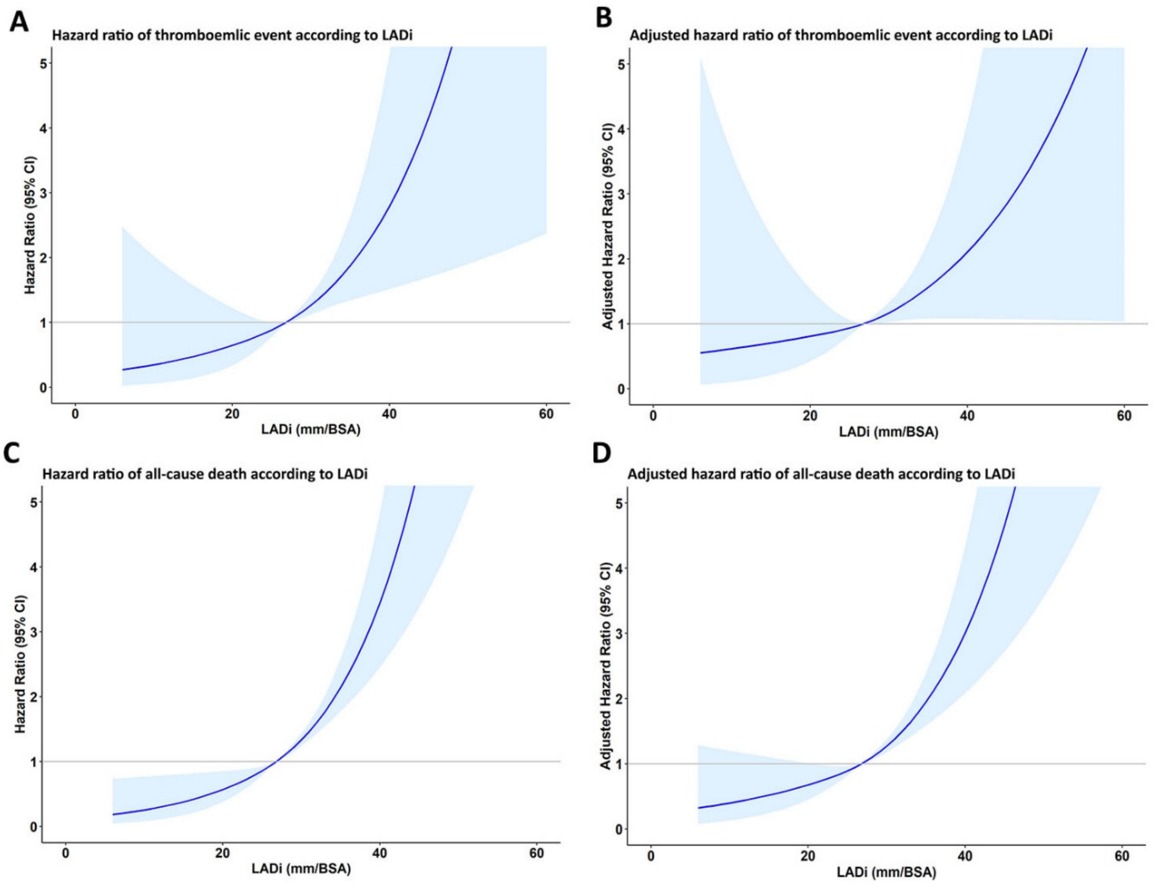

Supplementary Table S1. Incidence rate of thromboembolic event, all-cause death, and thromboembolic event or all-cause death according to left atrial diameter index (LADi) classification by the American Society of Echocardiography

LADi groups	Number of patients	Number of events	100 person-years	Rate per 100 person-years
**Thromboembolic event**				
LADi – normal to mildly abnormal	1184	32	32.02	0.99 (0.68–1.41)
LADi - moderately abnormal	447	17	11.66	1.46 (0.85–2.33)
LADi - severely abnormal	620	41	15.20	2.70 (1.94–3.66)
Total	2251	90	58.88	
**All-cause death**				
LADi – normal to mildly abnormal	1184	80	32.23	2.48 (1.97–3.09)
LADi - moderately abnormal	447	48	11.80	4.07 (3.00–5.39)
LADi - severely abnormal	620	119	15.37	7.74 (6.41–9.26)
Total	2251	247	59.40	
**Thromboembolic event or all-cause death**				
LADi – normal to mildly abnormal	1184	103	31.98	3.22 (2.63–3.91)
LADi - moderately abnormal	447	60	11.63	5.16 (3.94–6.64)
LADi - severely abnormal	620	145	15.14	9.57 (8.08–11.27)
Total	2251	308	58.74	

LADi – normal to mildly abnormal = LA diameter/BSA (cm/m^2) < 2.7

LADi – moderately abnormal = LA diameter/BSA (cm/m^2) 2.7–2.9

LADi – severely abnormal = LA diameter/BSA (cm/m^2) ≥ 3.0

Supplementary Figure S2. Bar graphs of Incidence rate of thromboembolic event (A), all-cause death (B), and thromboembolic event or all-cause death (C) when categorized left atrial diameter index (LADi) according to the classification by the American Society of Echocardiography into severely abnormal compared to the rest of the group.

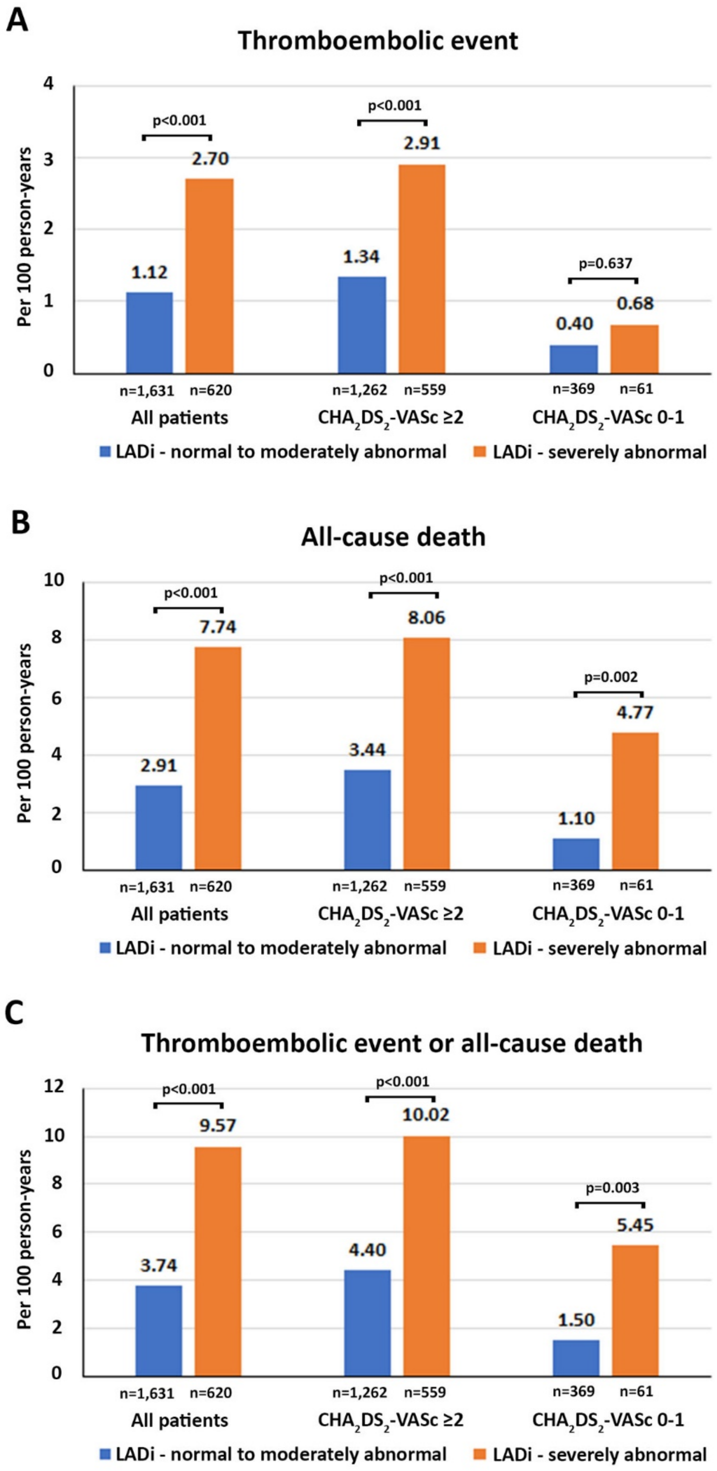

Supplement: Supplementary file 1 [file jcm-11-01838-s001.zip › jcm-1627792-supplementary.pdf]
